# Microbiota acquisition and transmission in *Drosophila* flies

**DOI:** 10.1016/j.isci.2023.107656

**Published:** 2023-08-17

**Authors:** Robin Guilhot, Anne Xuéreb, Auxane Lagmairi, Laure Olazcuaga, Simon Fellous

**Affiliations:** 1 CBGP, INRAE, CIRAD, IRD, Montpellier SupAgro, University of Montpellier, 34000 Montpellier, France; 2 Department of Agricultural Biology, Colorado State University, Fort Collins, CO 80523, USA

**Keywords:** Ecology, Microbiome, Model organism

## Abstract

Understanding the ecological and evolutionary dynamics of host-microbiota associations notably involves exploring how members of the microbiota assemble and whether they are transmitted along host generations. Here, we investigate the larval acquisition of facultative bacterial and yeast symbionts of *Drosophila melanogaster* and *Drosophila suzukii* in ecologically realistic setups. Fly mothers and fruit were major sources of symbionts. Microorganisms associated with adult males also contributed to larval microbiota, mostly in *D. melanogaster*. Yeasts acquired at the larval stage maintained through metamorphosis, adult life, and were transmitted to offspring. All these observations varied widely among microbial strains, suggesting they have different transmission strategies among fruits and insects. Our approach shows microbiota members of insects can be acquired from a diversity of sources and highlights the compound nature of microbiotas. Such microbial transmission events along generations should favor the evolution of mutualistic interactions and enable microbiota-mediated local adaptation of the insect host.

## Highlights

• Fly larvae acquire microbial symbionts from their mothers and the fruit substrate
• Adult males-associated microorganisms also contribute to larval microbiota
• Larvae-associated yeasts can persist through fly life cycle and over generations
• Transmission rates vary greatly among microbial strains

## Graphical abstract

**Figure:**
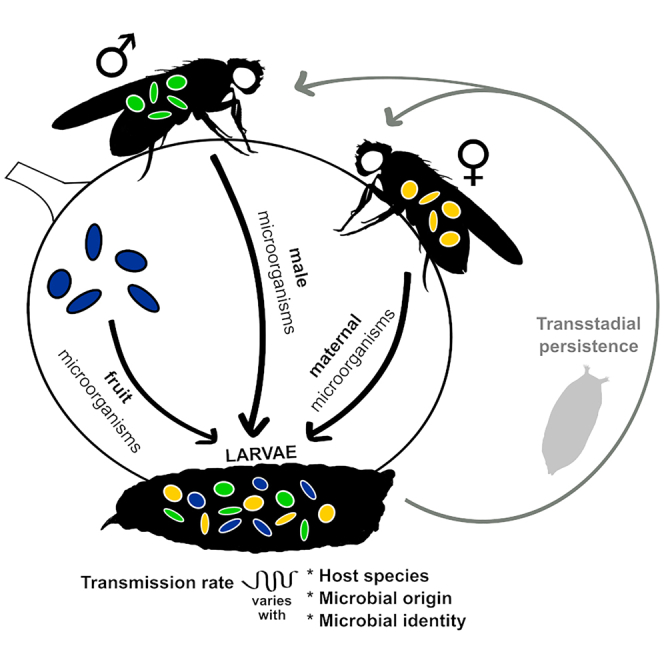

## Introduction

Insects, like most multicellular organisms, commonly host a diversity of facultative microorganisms. While the development of molecular methods facilitates describing insect microbial community composition,^1^ understanding how insects and facultative microorganisms associate often remains a challenge. Recent studies on this issue have shown that these associations are shaped by biotic and abiotic factors,^2^ influencing the way microorganisms are acquired by insect hosts.^3^^,^^4^^,^^5^^,^^6^ Microbial symbionts can indeed be inherited from the insect parents (vertical transmission, a phenomenon not restricted to intracellular symbionts), acquired from unrelated congeners (horizontal transmission), or acquired from another source (e.g., from insect substrate or environment).^7^ Many symbionts often display a combination of these transmission strategies.^8^ Since insects can vector microorganisms between natural habitats, microbial persistence along insect life and microbial transmission among individuals or generations further influence the dispersal of microorganisms among resources patches.^9^ Over longer time scales, the routes of symbiont acquisition and transmission by hosts influence the evolution of the effects each has on the other.^10^ For example, it is often expected that vertically transmitted microorganisms are more likely to have beneficial effects on their hosts than horizontally transmitted ones.^11^^,^^12^ Studying how insects acquire microbial symbionts will therefore better our understanding of the spatiotemporal dynamics of microorganisms among hosts and resource patches^13^ and our understanding of the evolution of symbiotic associations.^4^

Among insects, *Drosophila* flies that feed on decaying organic matter cohabit with a variety of extracellular bacteria and yeast that may colonize their digestive tract, their cuticle, and their nutritive substrate.^14^^,^^15^ Such microorganisms affect fly larva and adult nutrition, physiology, development, and behavior.^16^^,^^17^^,^^18^ Fly larvae affect microbial multiplication within the larval substrate while flying adults spread microorganisms among resource patches.^19^^,^^20^^,^^21^ *Drosophila melanogaster* is extensively used as a convenient model to understand the mechanisms of host-microbiome interactions^22^^,^^23^ because its bacterial microbiota is simple and easy to maintain under artificial conditions. However, how *Drosophila* flies associate with microorganisms under natural conditions remains poorly known. Several pioneering studies reported elements on the origin of extracellular microorganisms associated with *Drosophila* larvae and the persistence of such microorganisms through fly life cycle.^24^^,^^25^^,^^26^^,^^27^^,^^28^^,^^29^^,^^30^^,^^31^^,^^32^^,^^33^ Recently, two studies have shown stability and resilience to perturbation in bacterial microbiota members can occur,^32^^,^^34^ challenging the more common view that the *Drosophila* microbiota is unstable, requiring constant replenishment of symbionts through nutrition (see^22^). Yet, most of the available data were obtained under artificial conditions, for example by studying laboratory-adapted microorganisms, using artificial substrates, or working in absence of microbial competition. These types of experimental choices could cause misleading extrapolations of symbiont acquisition and transmission phenomena in the wild, as shown by Winans and colleagues.^35^ Here, we used fresh fruits, bacteria, and yeasts mainly isolated from the wild, and two *Drosophila* species of major interest but with contrasting ecology—the model organism *D. melanogaster* and the invasive pest *D. suzukii*—to conduct a series of assays investigating the origin and transmission of microorganisms associated with *Drosophila* larvae under ecologically realistic conditions. We first explored the contribution of adult females and males to larval microbiota. We then tested how yeast species, which we first associated to larvae, persisted throughout the fly life cycle and over generations.

## Results

### *Drosophila* females transmit symbionts to their offspring

**Figure 1:**
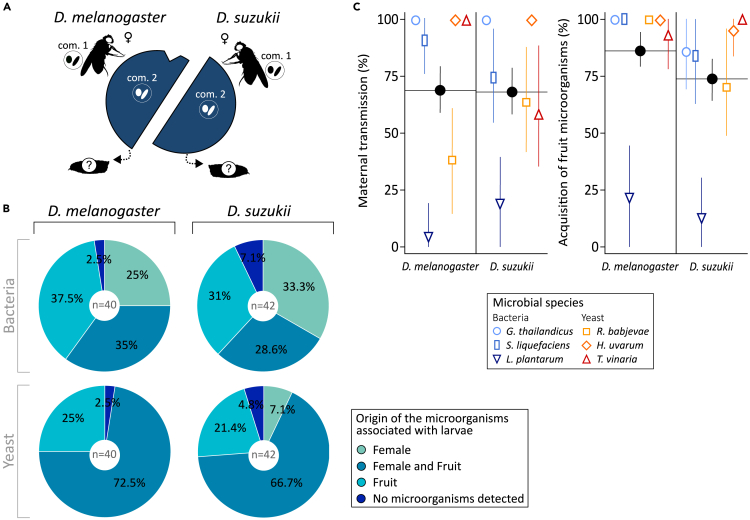
*Drosophila* larvae associated with maternal microorganisms and fruit microorganisms (A) Experimental setup. A mature female associated with a certain bacteria-yeast assemblage (*Com. 1*) was left to oviposit on a blueberry inoculated with a different bacteria-yeast assemblage (*Com. 2*). Fruits presented to *D. melanogaster* were wounded near the peduncle insertion, not in the case of *D. suzukii*. (B) Origin of the microorganisms associated with the larvae (% larval samples). (C) Proportion of microorganisms associated with mothers or fruit acquired by larvae among fly and microorganism species (% larval samples). Black dots indicate overall mean and 95% CI per *Drosophila* species (i.e., independent of microorganism species) while open symbols and related bars indicate mean and 95% CI for each microorganism (independently of the identity of the co-inoculated microorganism and the identity of their competitors in the experimental system; sample sizes n are given in Table S9). Confidence intervals were calculated using normal approximation method.

Previous studies showed that *Drosophila* flies, like other insects,^36^ can exhibit maternal transmission of microbiota, at least under laboratory conditions.^24^^,^^26^^,^^27^^,^^32^^,^^33^ On this basis, we hypothesized that larvae may also inherit female-associated microorganisms under ecologically realistic conditions, i.e., when eggs are deposited in fresh fruit inoculated with other microorganisms (hereafter referred as fruit microorganisms). We also expected that whether or not larvae acquired a given microorganism would depend on the microbial species and that of the fly, as well as the transmission route, i.e., from mothers or fruit surface (hereafter referred as microbial origin). We expected the different ecologies of *D. melanogaster* and *D. suzukii* would affect how their larvae acquire their symbionts. *D. melanogaster* prefers to oviposit on damaged or overripe fruit substrates potentially rich in microorganisms while *D. suzukii* is known for its preference to lay eggs on ripe and ripening, pristine fruit substrates.^37^^,^^38^ We confirmed these species-specific behavioral differences with a preliminary experiment in mesocosms with laboratory and wild-caught flies (Figure S1). Since *D. suzukii* females insert their eggs deep into the fruit flesh, their young larvae should primarily recruit microorganisms deposited by the mother during oviposition rather than those present on the surface of the fruit. To test our predictions, sexually mature females of both species associated with a certain bacteria-yeast community (each composed of one yeast and one bacterial strain, *Com. 1* in Figure 1A) were individually left to oviposit for 24 h on a blueberry previously surface-inoculated with a different bacteria-yeast community (*Com. 2* in Figure 1A). These different microorganisms were chosen among the six strains used in our study: three yeast (*Rhodotorula babjevae*, *Hanseniaspora uvarum*, *Trigonopsis vinaria*) and three bacteria (*Gluconobacter thailandicus*, *Serratia liquefaciens*, *Lactobacillus plantarum*). Five days after fruit exposure to females, larvae were collected from fruit, crushed, and plated on selective growth media tuned to distinguish the different microorganisms previously inoculated to mothers or fruit surface. The different metabolic abilities of the four microorganisms chosen (i.e., two in females, two on fruit) indeed enabled their recognition once plated (see STAR Methods for a presentation of these microorganisms and a description of our recognition method).

In most cases, *Drosophila* larvae harbored maternal microorganisms, alone or in combination with fruit microorganisms (Figure 1B). The larval acquisition rate of each microorganism per microbial origin and fly species is presented in Figure 1C. Larval acquisition from fruit (80% acquisition – 95% CI [74,86]) was slightly more frequent than from mothers (68% acquisition – 95% CI [61,75], p = 0.0142) (Table S2). Note these two pathways are not mutually exclusive as larvae could harbor symbionts from both origins. Contrary to our expectation, the frequencies of maternal transmission and acquisition from fruit did not differ markedly among fly species (see the marginally non-significant interaction between *Microbial origin* and *Fly species* in Table S2). Some microbial species were associated with larvae more frequently than others (Table S2; Figure S2). For example, the bacterium *Gluconobacter thailandicus* and the yeast *Hanseniaspora uvarum* were recovered more often from larval homogenates than the yeast *Rhodotorula babjevae* (Figure S2). In addition, larvae rarely acquired the laboratory-isolated bacterium *Lactobacillus plantarum* compared to all other microbial species (Figure S2). It should be noted that our data do not provide estimates of individual transmission rates from mothers to offspring as we crushed together groups of larvae retrieved from the same fruit piece. Although it could be expected that all larvae that share the same substrate share the same microbiota, previous reports on *Drosophila* adults have shown that the microbiota of individuals reared in the same conditions can differ.^34^ The positive association between the number of larvae in the samples and the likelihood of microorganism detection (Table S2) may result from two alternative phenomena. First, it could be due to microbiota heterogeneity among cohabiting larvae: a greater number of larvae in a sample would lead to a greater number of microorganisms sampled. Second, it is also possible that increasing the number of larvae in a single piece of fruit would increase the probability each microorganism is recruited by and later shared among larvae of the same fruit. Independent from these considerations, our results establish that in natural set-ups *D. melanogaster* and *D. suzukii* larvae not only associate with yeast and bacteria from their surrounding environment but also with microorganisms transmitted from their mothers.

### *Drosophila* males transmit symbionts to larvae developing in their territory

**Figure 2:**
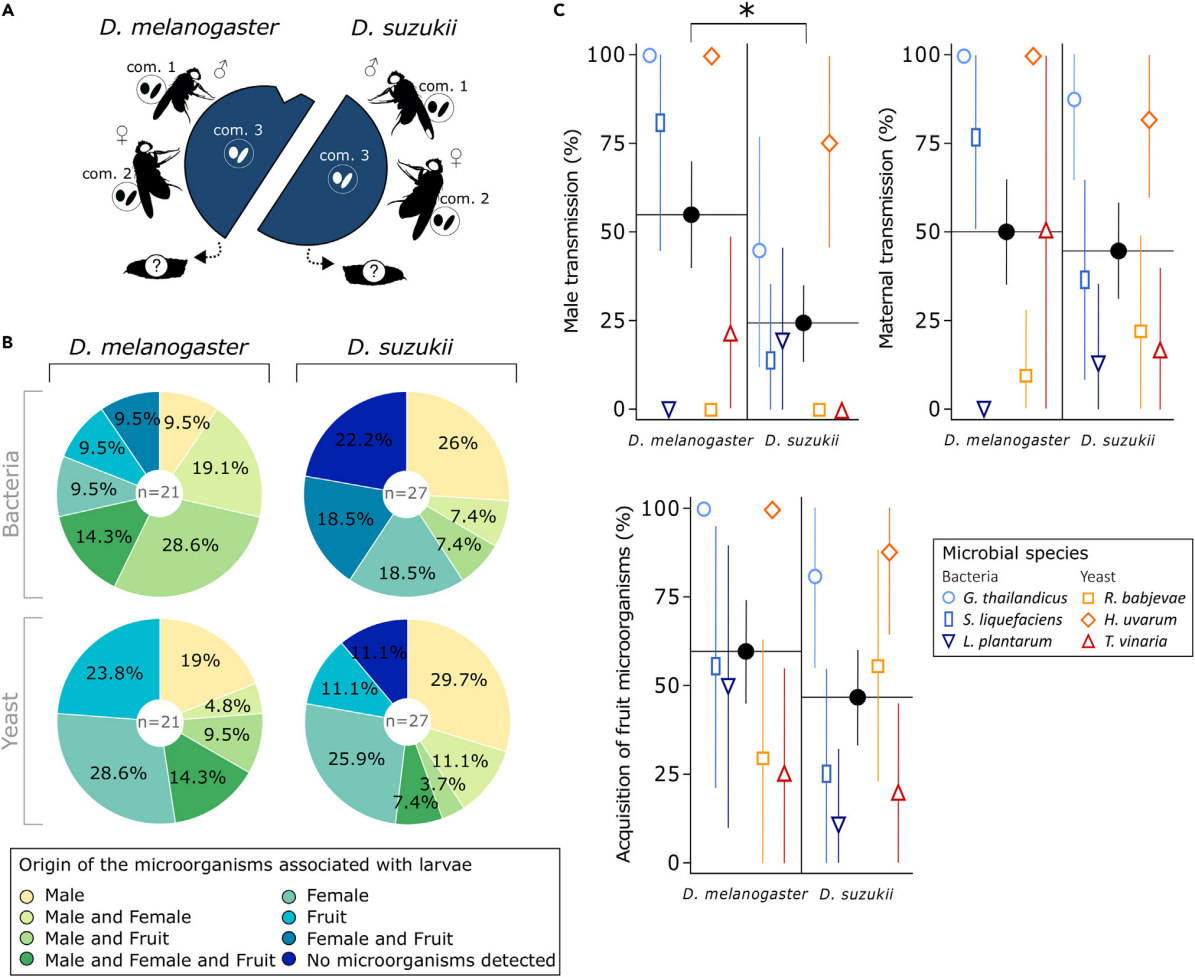
*Drosophila* larvae associated with microorganisms from adult males (A) Experimental setup. A mature male and a mature female associated with two different bacteria-yeast assemblages (*Com. 1* and *Com. 2*) were released in a cage that contained a blueberry inoculated with a third bacteria-yeast assemblage (*Com. 3*). Fruits presented to *D. melanogaster* were wounded near the peduncle insertion, not in the case of *D. suzukii*. (B) Origin of the microorganisms associated with the larvae (% larval samples). (C) Proportion of microorganisms associated with males, mothers, or fruit acquired by larvae among fly and microorganism species (% larval samples). Black dots indicate overall mean and 95% CI per *Drosophila* species (i.e., independent of microorganism species) while open symbols and related bars indicate mean and 95% CI for each microorganism (independently of the identity of the co-inoculated microorganism and the identity of their competitors in the experimental system; sample sizes n are given in Table S9). Confidence intervals were calculated using normal approximation method. The asterisk “∗” indicates a significant difference (α = 0.05, generalized linear mixed model with binomial distribution and logit link function).

While vertical transmission from the mother and environmental acquisition are important routes for the acquisition of microorganisms in *Drosophila* larvae (Figure 1), horizontal transmission—i.e., from unrelated conspecific individuals—could also drive the microbiota composition. Field and laboratory observations revealed that *Drosophila* males were frequently present on fruits that females use for oviposition (Figure S3). *D. melanogaster* males can be territorial, defend oviposition sites, and form leks.^39^^,^^40^ We confirmed with a preliminary experiment that *Drosophila* males deposit viable microbial inoculates on fruit surface (Figure S4). We hence hypothesized that the microorganisms associated to *Drosophila* males would contribute to larval microbiota through the deposition of microbial cells when males roam on oviposition sites. Moreover, we expected greater male transmission from *D. melanogaster* males than *D. suzukii* males: unlike *D. suzukii* males, *D. melanogaster* males are present on fruit wounds, where females preferentially oviposit and microorganisms are more likely to grow than on fruit skin. In a new experiment, we tested whether *Drosophila* males transmitted their microbial symbionts to offspring of conspecific females (Figure 2A). To this end, a mature male and a mature female were released in a cage that contained a fresh blueberry following a set-up similar to the experiment described in the previous section. Male, female, and fruit were all associated with a different bacteria-yeast community (i.e., three different pairs of bacteria and yeast among the six strains used in our study, see *Com. 1*, *2*, and *3* in Figure 2A). Five days after fruit exposure to adults, larvae were collected from the fruit, crushed, and plated on selective growth media tuned to identify the nature of the associated microorganisms based on their metabolic abilities.

Numerous larvae contained microorganisms from males, alone or in combination with fruit and/or female microorganisms (Figure 2B). Male transmission of extracellular microorganisms is in general less frequently reported that maternal transmission in insects.^41^^,^^42^ In a previous study, Pais and colleagues^32^ studied the symbiotic association between *D. melanogaster* and *Acetobacter thailandicus*, a bacterium isolated from wild flies that accelerates larval development. They showed that *Drosophila* males and females associated with this bacterial strain produce offspring that develop faster on artificial diet than larvae issued by adults free from *A. thailandicus*, indirectly suggesting that adults—including males—could transmit *A. thailandicus* to their offspring. To our knowledge, our observations constitute the first direct evidence that *Drosophila* males transmit extracellular microorganisms to larvae in an ecologically realistic context, with co-occurring microorganisms and natural fruit substrate. The larval acquisition rate of each microorganism per microbial origin and fly species is presented in Figure 2C. Larvae acquired male microorganisms (38% acquisition – 95% CI [28,47]) less frequently than they acquired fruit microorganisms (52% acquisition – 95% CI [42,62], p = 0.0126) but not less frequently than female microorganisms (47% acquisition – 95% CI [37,57], p = 0.1807) (Figure 2C, Table S5). As predicted, male transmission was more frequent for *D. melanogaster* (55% of samples – 95% CI [40,70]) than for *D. suzukii* (24% of samples – 95% CI [13,35]) (Figure 2C, Table S5). Larval acquisition of microorganisms varied among microbial species (Table S5; Figure S5), independent of their origin but with slight differences among fly species (Table S5). Interestingly, comparing larval acquisition of the different microbial species in the first experiment (previous section) and the second experiment (present section)—excluding male microorganisms (see STAR Methods for statistical details)—reveals that three microbial species were more frequently picked by larvae in the first experiment than in the second (Table S6; Figure S6). This shows microbial colonization success depends on fine-scale ecological context.

With data from the same experiment, we attempted to explain how males transmit microorganisms to larvae. Based on our preliminary observations (Figure S4), we expected a correlation between the time spent by individual males on fruit surface (determined as the proportion of visual observations with male presence on the fruit oviposition site over 24 h) and the likelihood of male transmission. It was, however, not the case in the present experiment (Table S5). Alternatively, male transmission may have relied on sexual transmission during mating, a frequent phenomenon in insects^43^^,^^44^^,^^45^ that has been reported in *Drosophila* flies.^25^^,^^46^ We therefore tested whether mating during our experiment explained male symbiont transmission success. Mating was rarely observed (33% of *D. melanogaster* couples mated, 7% for *D. suzukii* couples) and this factor had no significant influence on male symbiont detection in larvae (Table S5). As neither time spent on fruit oviposition site nor mating occurrence explained male symbiont transmission, the mechanism of this phenomenon remains unknown.

Larval acquisition of microorganisms transmitted by males should influence the evolution of microbial effects on male hosts. It is widely assumed that vertical transmission selects symbionts toward higher benevolence (or lesser costs) to their hosts when compared to horizontal transmission.^11^^,^^12^ In our experiment, as in the field, transmission of male microorganisms was not strictly vertical since it was not contingent upon male reproduction. Indeed, even in the absence of mating with the females, males transmitted their symbionts to larvae. Another mechanism selecting for beneficial effects of symbionts on their male hosts may, however, occur. In *D. melanogaster*, the largest males more successfully defend oviposition sites than the smallest ones, which fail at securing a territory.^47^^,^^48^ Large or more vigorous males that better defend oviposition sites could therefore transmit their microorganisms to larvae more frequently than weak males. Microorganisms associated to male are therefore selected for improving host health and boosting size. Since the size of an adult fly is largely determined by its larval conditions,^49^^,^^50^ the previous scenario necessitates symbionts of male larvae maintain during metamorphosis and adult life until they can be transmitted, a phenomenon we investigated in the experiment described in the following section.

### Yeast associated with larvae maintain through *Drosophila* metamorphosis, persist throughout adult life, and are transmitted to offspring

**Figure 3:**
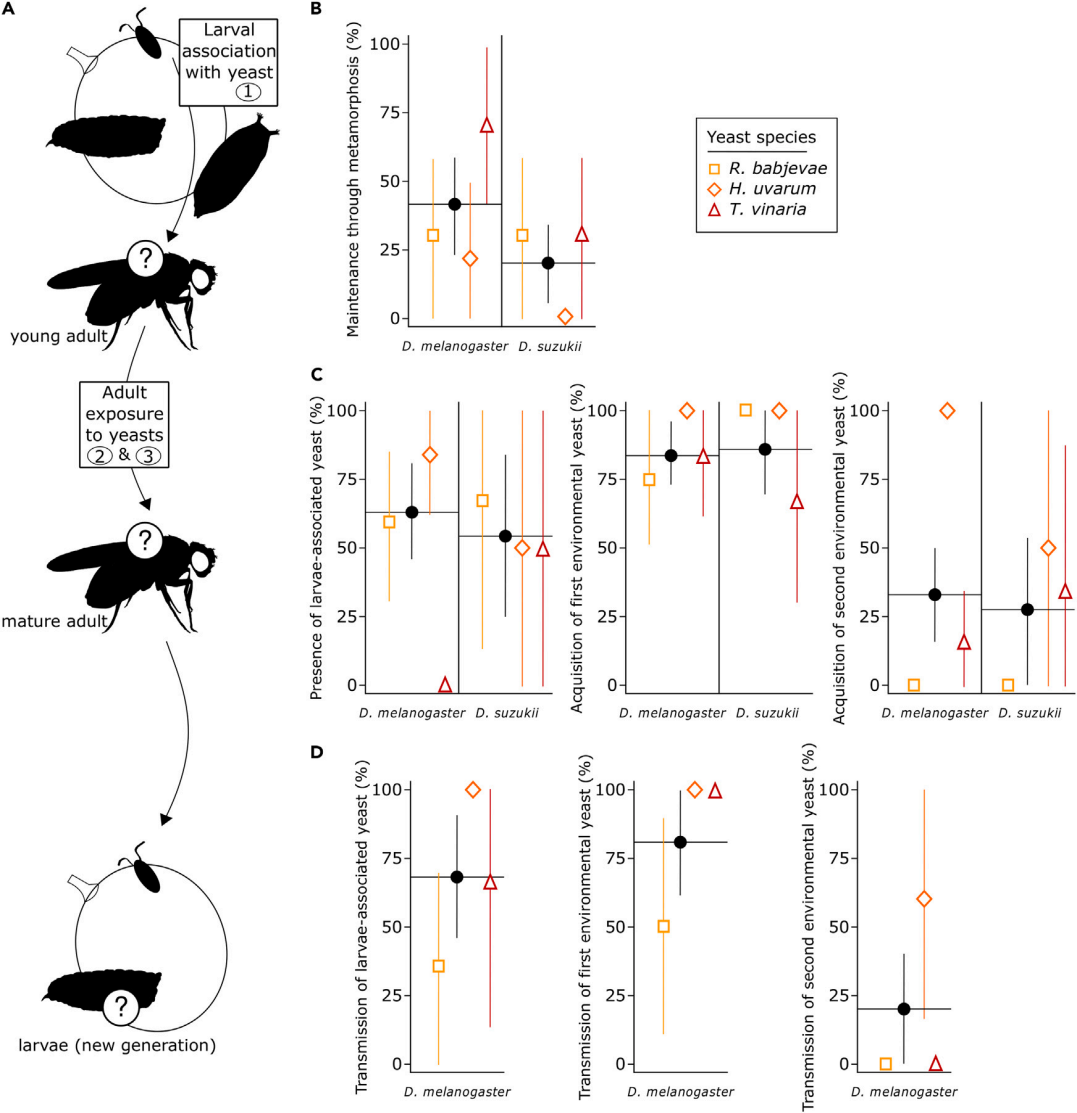
Larvae-associated yeasts persisted through *Drosophila* life cycle and over generations (A) Schematic of the experimental setup. (B) Maintenance of yeasts through *Drosophila* metamorphosis among fly and yeast species (% young adult samples). (C) Presence of larvae-associated yeasts and acquisition of environmental yeasts in mature adults among fly and yeast species (% mature adult samples). (D) Transmission of the different adult-associated yeasts to a new *D. melanogaster* generation (% larval samples). In Figures (A), (B), and (C), black dots indicate overall mean and 95% CI per *Drosophila* species (i.e., independently of the yeast species and the fly sex) while open symbols and related bars indicate mean and 95% CI for each yeast (independently of the identity of the other yeast(s) inoculated in the experimental system; sample sizes n are given in Table S9). Confidence intervals were calculated using normal approximation method.

Several studies reported that microorganisms associated with *Drosophila* larvae can be detected in young adults, a phenomenon called transstadial maintenance or maintenance through metamorphosis.^24^^,^^25^^,^^28^^,^^51^^,^^52^ In the wild, environmental transmission between the larval and adult stages is unlikely, because *Drosophila* juveniles usually leave larval substrates to pupate elsewhere, often in soil.^53^^,^^54^ We mimicked this behavior in an additional experiment to confirm that yeasts associated with *Drosophila* larvae maintained until adult emergence under ecologically realistic conditions. We further hypothesized the frequency of this phenomenon would vary among microbial species, fly species, and fly sex. The experiment started by associating fungus-free eggs with a single yeast strain in a surface-sterilized grape berry (Figure 3A). Several days later, when pupae formed, the contaminated fruit piece was removed from the container. Later, adults from these pupae were aseptically collected within a few hours after emergence. The procedure was designed to ensure microorganisms retrieved from emerging adults were those present in or on the pupa, it is nonetheless plausible minute cage contamination occurred during larval dispersal at the wanderer stage. The yeast strain that was inoculated to larval fruit was detected in about 41% and 20% of the *D. melanogaster* and *D. suzukii* adults, respectively (Figure 3B), this difference was not significant (χ^2^ = 2.07, df = 1, p = 0.1503). Larval yeasts were marginally more frequently retrieved from young females (41% acquisition – 95% CI [23,59]) than males (20% acquisition – 95% CI [6,34]) (χ^2^ = 3.98, df = 1, p = 0.0461); yeast species only had a marginally non-significant effect (χ^2^ = 4.99, df = 2, p = 0.0826).

The following step was to investigate whether symbionts of young adults that originated from the larval substrate remained associated further in the life cycle and could be transmitted to offspring. We hence prolonged the previous experiment using a subset of the adults produced. These adults were not maintained in an aseptic environment as in most comparable studies, instead we exposed them to other, potentially competing microorganisms (Figure 3A). More precisely, freshly emerged adults, which potentially still carried the yeast strain acquired from their larval stage, were released in cages that contained a grape berry inoculated with another yeast strain. This berry was left for five days in the cage until it was replaced by another berry inoculated with a third yeast strain. Two days later, the berry was removed, and flies were offered to oviposit on a surface-sterilized grape berry. The yeast strains present in these adults were then analyzed, along with those present in the larvae we retrieved from the surface-sterilized grape berries in which the adults had laid eggs. This revealed the presence of larval yeasts in mature adults (Figure 3C) and therefore transstadial maintenance. Larval yeasts were also found in the larvae of the following generation, meaning parents transmitted the yeasts they were initially associated with at the larval stage (this information is only available for *D. melanogaster* as too few *D. suzukii* larvae were sampled to conclude; Figure 3D). Such maintenance of larval symbionts, after metamorphosis and over adult life, may have occurred by two non-excluding mechanisms. First, larval symbionts could colonize the adult fly in a stable fashion. Two recent independent studies show wild isolates of the bacteria *Lactobacillus plantarum* and *Acetobacter thailandicus* durably colonize the first gut region of the host despite the continuous ingestion of other microorganisms.^32^^,^^34^ Second, young adults could have inoculated the fruit berries present in the cage^55^ with the larval yeast they contained. These microorganisms would have multiplied in fruit flesh, mixing with the other yeast strain previously inoculated there, and later be re-ingested by foraging adults. Unfortunately, we did not monitor microbial community composition in the berries the adults were exposed to, rejecting one of these two hypothetical processes is thus not possible with our data. It is nonetheless remarkable that larval yeast strains were largely detected in mature adults despite the successive exposure to two additional strains over a seven-day period (Figure 3C, Table S7). Moreover, old adult flies contained the second environmental yeasts they were exposed to less frequently than the first (χ^2^ = 13.98, df = 1, p = 0.0002) (Figure 3C). This difference could be interpreted as a form of precedence effect, at the advantage of symbionts that colonize the host first, as described during the stable colonization of the adult *Drosophila* gut by bacteria.^34^ It could also be the result of an exposure time too short for the second environmental yeast to outnumber the yeast previously acquired, adult exposure time to the first environmental yeast (five days) was indeed longer than the exposure time to the second environmental yeast (two days). This experiment suggests that, under ecologically realistic conditions, yeasts associated with *D. melanogaster* and *D. suzukii* larvae can maintain throughout metamorphosis and that, despite host exposure to other microorganisms during adult life, yeasts associated with *D. melanogaster* larvae can be transmitted to the following generation.

## Discussion

We observed that both females and males of two *Drosophila* species with contrasting ecologies transmitted the bacteria and yeasts of their microbiota to larvae. Yeast strains associated to larvae further remained associated with their host throughout metamorphosis and adult life, until they were transmitted to the progeny. These results, obtained under ecologically realistic conditions and using microorganisms isolated from the wild, show a diversity of colonization paths and support lasting associations between microbiota members and *Drosophila* flies in the wild.

### Multiple processes explain microbiota assembly

This study shows that microbiota members of insect model organisms such as *Drosophila* flies can be acquired from a diversity of sources. Environmental acquisition and vertical transmission from the mother were both pervasive (Figure 1). Male contribution varied among host species (Figure 2). Different strains of yeasts and bacteria also exhibited contrasted transmission potentials. These observations show the compound nature of microbiotas, which result from several ecological and evolutionary processes embedded in single host generations and unfold over larger time scales (Figure 3).

The multiple processes that lead to microbiota assembly—and its association with host genome—could lead to interactions and even conflicts between microbes and/or host genes.^56^ Here, it may be the case for male transmission of symbionts to juveniles, a phenomenon that has been described in several systems.^41^^,^^42^ The larvae that developed in fruit exposed to males often acquired their yeasts and bacteria (Figure 2), even in absence of mating with the female (Table S5). Subsequent larvae hence received their genes and symbionts from different males. This original feature may lead to conflicts between agents of biological information, such as genes and symbionts.^56^^,^^57^ In addition, other transmission conflicts may occur between larva-associated microorganisms which paths cross following their acquisition from the environment or from adults. Indeed, maximizing the fitness of each microorganism may require different host phenotypes or different allocations of limited resources. Interactions and conflicts between microbiota members have been described in a variety of systems.^58^ In *Drosophila melanogaster*, for example, the transstadial maintenance of larval yeast symbionts depends on the nature of co-occurring bacteria.^52^ The regular shuffling of microbiota composition among individual hosts, and the diverse transmission paths exhibited by microbial community members, assuredly set the stage for an array of adaptations to tune microbial phenotypes and strategies to conditions met in the host and/or in its surrounding environment.

### Evolutionary consequences of parent to offspring transmission

Although parent to offspring transmission was not the only process of microbiota assembly, its high prevalence has clear evolutionary consequences. Evolutionary theory predicts mutually beneficial interactions evolve when the traits and strategies that favor host fitness also benefit symbionts.^8^^,^^11^^,^^59^ The transmission of microbiota members from females to their offspring, as described in the present study, creates conditions selecting for beneficial effects of microorganisms on hosts. In *Drosophila* flies, this could rely on the nutrients provided by extracellular symbionts,^60^ their effects on host developmental strategy,^61^^,^^62^ on protection against pathogens, as described in other insects,^63^ or even on reproductive output.^64^ In addition, the persistence over generations of microbiota members with substantial effects on host phenotype can fuel host adaptation to local conditions,^65^^,^^66^ even though, in some conditions, local adaptation could also proceed through environmental acquisition of adequate symbionts. Vertical transmission makes possible hologenome selection and transmission over generations of combinations of host genes and symbionts.

Surprisingly, symbiont transmission patterns were very similar in *D. melanogaster* and *D. suzukii* despite their different ecologies (Figure S1) no matter the microbial species considered. We cannot exclude these observations are due to imperfect microcosm conditions or to the specific genotypes of flies we used. It could also show that variation among microbiota members have a stronger influence on symbiont transmission than host ecology. Nonetheless, the two fly species differed in terms of male transmission (greater in *D. melanogaster* than in *D. suzukii* – Figure 2C), an observation that implies greater selection for beneficial effects of symbionts on their host in *D. melanogaster* than in *D. suzukii*.

### Implications for microbial dynamics in space and time

Orchards, shrubs, and cities where *Drosophila* and their microbial symbionts occur embody the very definition of meta-populations: fruits are ephemeral patches of finite resources among which dispersal is a necessity. Many of the yeast and bacterial species found in insect microbiota are facultative symbionts; their vectoring by hosts to new fruit pieces would be the major benefit gained from the association.^55^^,^^67^^,^^68^^,^^69^ Flies may transmit microorganisms to eggs and juveniles (Figures 1 and 2), or inoculate suitable substrates devoid of larvae, such as fruit wounds. In the latter case, some strains of the yeast *Saccharomyces cerevisiae* attract *Drosophila* adults better than others, they are the ones that transmit to pristine substrates best.^55^

In our experiments, transmission to eggs and juveniles varied markedly among the microbial species assayed. For example, the yeast *Hanseniaspora uvarum*, known to attract *Drosophila* adults^70^^,^^71^^,^^72^^,^^73^ and frequently found associated with them,^15^^,^^74^^,^^75^ was often transmitted to larvae (Figure S2; Figure S5). However, it did not seem to maintain well through *Drosophila* metamorphosis (Figure 3B). By contrast, the yeast *Trigonopsis vinaria* was less frequently acquired by larvae than *H. uvarum* in one of the experiments (Figure S5) but maintained well throughout fly metamorphosis (Figure 3B). Although it is not possible to generalize with a handful of microbial isolates, our data suggest that wild microorganisms may vary in their strategies of fly-mediated dispersal.^76^ Some species, like *H. uvarum*, may be better at attracting adults while others, like *T. vinaria*, may be better at transmitting between life stages or to offspring. These strategies could reflect the ecology and physiology of these two microorganisms. *H. uvarum* is frequently isolated from the surface of fruits reaching adequate maturity.^77^ The strain of *T. vinaria* used in our experiment was isolated from fly ovaries. Colonizing insect tissue seems a good strategy to improve chances of maintenance during host metamorphosis.

Differences among microbial strains may also reflect their recent evolutionary history. In both *D. melanogaster* and *D. suzukii*, the bacterium isolate of *Lactobacillus plantarum* used in our experiment showed poor transmission rates (Figures 1C and 2C). This strain was the only one in our experiment that originated from a laboratory colony where selective pressures for transmission are suppressed by the housing of flies in small vials where adults and their progeny share the same substrates. In these set-ups, it was shown that adaptation to nutritive medium composition, rather than to the host, can drive bacterial effect on *Drosophila* phenotype.^31^

The processes behind host microbiota assembly and the dynamics of facultative symbionts in space and time are two faces of the same coin. In both cases, unveiling how and why different microorganisms have different transmission strategies and effects on hosts will necessitate mechanistic insights and a fine description of the selective pressures they are under.

### Limitations of the study

• The study was not designed to unravel microbial interactions and their potential effects on the phenomena we described.
• Further research is needed to unveil the mechanisms behind stable associations between adults and symbionts, for example to discriminate between constant microbial replenishment from the feeding substrate and long-lasting microbial colonization of the internal parts (or external) of the insect.
• In two out of three experiments, microbe transmission was assessed from groups of larvae collected in the same fruit piece, not from individuals.

## STAR★Methods

### Key resources table

**Table:** 

REAGENT or RESOURCE	SOURCE	IDENTIFIER
**Bacterial and virus strains**		

*Gluconobacter thailandicus*	Isolated from a fly-infested grape berry^78^	N/A
*Serratia liquefaciens*	Isolated from *D. suzukii* ovaries^78^	N/A
*Lactobacillus plantarum*	Isolated from *D. melanogaster*^79^	GenBank: EU096230

**Other**		

*Rhodotorula babjevae*	Isolated from a fly-infested grape berry^62^	GenBank: MN684819.1
*Hanseniaspora uvarum*	Isolated from *D. melanogaster* feces^62^	GenBank: MN684824.1
*Trigonopsis vinaria*	Isolated from *D. suzukii* ovaries^62^	GenBank: MN684816.1

### Resource availability

#### Lead contact

All requests for additional information and resources should be directed to Robin Guilhot (guilhoro@gmail.com).

#### Materials availability

This study did not generate new unique reagents.

### Experimental models and study participants

#### Drosophila stocks

Two main *D. suzukii* strains were founded from wild individuals collected in 2013 in Gaujac, Southern France (population A) and from wild individuals collected in early 2018 around Avignon, Southern France (population B), respectively. A *Drosophila melanogaster* strain was founded from wild individuals collected in late 2017 that emerged from a pomegranate from the area of Montpellier, Southern France (population C). All colonies were maintained at 21°C with 70% humidity and a 14 h photoperiod on a carrot-based diet (37.5 g.L^-1^ dried carrot powder (Colin Ingredients SAS), 37.5 g.L^-1^ sugar, 22.5 g.L^-1^ inactive dry yeast, 15 g.L^-1^ corn meal, 11.25 g.L^-1^ agar, 5 mL.L^-1^ propionic acid, 3.3 g.L^-1^ nipagin diluted in 2.5 mL.L^-1^ ethanol). None of the populations used in this study were screened for *Wolbachia* bacteria.

#### Microbial isolates

We used six different yeast (*Rhodotorula babjevae*, *Hanseniaspora uvarum*, *Trigonopsis vinaria*) and bacterial taxa (*Gluconobacter thailandicus*, *Serratia liquefaciens*, *Lactobacillus plantarum*), all of which had previously been reported in *Drosophila* flies and/or their environment (Supplemental information). Information related to each microbial strain (e.g., source, growth conditions) is provided in Supplemental information. Five strains were isolated from wild flies and fly-infested organic fruits in late 2017 and were molecularly identified by sequencing the ITS1 rDNA region (yeasts) and V4 16S rRNA region (bacteria) (Supplemental information). The sixth microorganism is an isolate of the bacterium *Lactobacillus plantarum* that was collected from *D. melanogaster*^79^ and is extensively used to investigate the underlying physiological mechanisms of *Drosophila* - microbiota associations in the laboratory (Supplemental information). To detect and discriminate between these microorganisms in biological samples collected during the experiments, we simultaneously plated subsamples (serially diluted) on up to five different solid culture media that were all appropriate to the growth of one or several microorganisms. These media were Glucose-based medium (GLU) (33.3 g.L^-1^ D-glucose, 33.3 g.L^-1^ ammonium sulfate, 11.3 g.L^-1^ yeast nitrogen base wo amino acids (BD Difco™), 15 g.L^-1^ agar), Galactose-based medium (GAL) (33.3 g.L^-1^ D-galactose, 33.3 g.L^-1^ ammonium sulfate, 11.3 g.L^-1^ yeast nitrogen base without amino acids (BD Difco™), 15 g.L^-1^ agar), Mannitol medium (MAN) and De Man, Rogosa and Sharpe medium (MRS). After incubation (for 48 h at 24°C for GLU, GAL and MAN, for 48 h at 30°C for MRS), microorganisms that eventually grew were distinguished according to the morphology of their colonies (shape, colour, transparency, and texture). PCR amplification and Sanger sequencing allowed to confirm repeatability and specificity of these colony properties under our experimental conditions, and therefore the robustness of our morphological identifications during reported experiments. Biological samples were systematically serially diluted and plated in triplicate, and cells were counted at the dilution deemed optimal to distinguish between different colonies based on their morphology (see above).

### Method details

#### Transmission of microorganisms to fly larvae

##### Gnotobiotic flies

Eggs of *D. suzukii* (population A) and *D. melanogaster* (population C) flies were collected on grape juice agar plates. To remove any extracellular microorganism present on and in the embryo outer envelope, the eggs were dechorionated using the protocol of Koyle and colleagues^80^ which relies on successive sodium hypochlorite and sterile water washes. Axenic colonies from dechorionated eggs were maintained at 23°C on a sterile banana-based diet (233 g.L^-1^ organic banana, 62 g.L^-1^ sugar, 62 g.L^-1^ inactive dry yeast, 10 g.L^-1^ agar). Five days before the experiments, tubes that contained axenic young adults (not separated by sex) were inoculated with 10 μL of a pure overnight bacterial culture and 10 μL of a pure overnight yeast culture (these microorganisms were chosen among the six used in our study, see previous Microbial isolates section). Slightly unscrewing the caps allowed the inoculation of the tubes without the need for CO_2_. Axenic or gnotobiotic status of each fly colony was systematically controlled by plating fly and diet samples (serially diluted) on appropriate solid growth media. The adult flies used in the two experiments described below were always transferred manually to the experimental containers using an aspirator tube with interchangeable and sterile tips and filters.

##### Fruit manipulation

Conventional blueberries were surface-sterilized following the protocol of Behar and colleagues.^81^ Berries were then inoculated in surface with a bacteria-yeast suspension (among the six microorganisms used in our study, see above). Fruits were dipped for 5 min in 20 mL of microbial cells from overnight cultures. These cultures were started using thawed, quantified microbial aliquots to reach a homogeneous initial cell concentration. Fruits were then suspended in sterile PBS (Phosphate-buffered saline), vortexed during the last 2.5 min, and air-dried during 18 h before their use in the experiments. In this way, approximately 5 000 microbial cells (and exceptionally 50 000 cells in the first experiment, see next section) of each microorganism were deposited on fruit surface. These concentrations were controlled by plating 5 fruit samples per microorganism (serial dilution) on suitable solid culture media. This number was determined based on estimates of microbial cell numbers deposited by insects on fruit surfaces conducted in our laboratory and not yet published. Presence and number of cells on fruit surface were controlled by plating fruit samples (serially diluted) on appropriate solid growth media: all microorganisms maintained at their initial cell concentration for at least five consecutive days.

Blueberries were always arranged with peduncle insertion upwards inside the experimental cages. This berry area was identified as an attractive oviposition site for *D. suzukii* females as they predominantly laid their eggs in skin cracks around the peduncle insertion in a preliminary experiment. To allow oviposition of *D. melanogaster* females, we slightly wounded the fruit surface near the peduncle insertion using a sterile pipette tip, only after microbial inoculation of the fruit surface, see above.

##### Assessing maternal transmission

A gnotobiotic female was released in a plastic, cylindrical sterile cage (Ø 4 cm, 8 cm high) that contained a blueberry inoculated on the surface with a different bacteria-yeast community (Figure 1A). For this experiment only, we evaluated whether the concentration of fruit microorganisms (i.e., 5 000 or 50 000 microbial cells on fruit surface) influenced their acquisition by the larvae, which was not the case (Table S2). A piece of sterile, hydrated cotton was added in the cage to avoid fly dehydration. After 24 h, the female was aseptically collected, crushed in sterile PBS + 20% glycerol for 2 min at 30Hz with two Ø3 mm sterile glass beads using a Tissue Lyser II (Qiagen), and stored at -80°C. After five days, up to ten larvae were aseptically collected from the fruit and crushed together in sterile PBS as described above. Larval homogenate subsamples (serially diluted) were plated on appropriate solid growth media to detect and discriminate larvae-associated microorganisms independently of their location inside or outside the larvae. Plating samples of surface-sterilized fruits not exposed to flies allowed us to confirm the absence of cultivable microorganisms from fruits, except very sporadic filamentous fungi. The experiment was conducted at 23°C with 65% humidity and a 12 h photoperiod. For each *Drosophila* species, we created a cage for every possible female-fruit microbial combination among the six microorganisms we studied, i.e., 36 combinations, for each of the two fruit concentrations, i.e., 36 ∗ 2 = 72 cages. However, some females did not lay eggs. Larvae were therefore collected from 40 cages for *D. melanogaster* and from 42 cages for *D. suzukii*.

Prior to this experiment, a preliminary trial was conducted to study *D. melanogaster* and *D. suzukii* egg laying preference using small ecologically realistic microcosms. *D*. *suzukii* adults were collected in a wooded area in Montferrier-sur-Lez, Southern France using vinegar baits or were obtained from a laboratory population founded with adults collected in Prades-le-Lez, Southern France. *D*. *melanogaster* adults were all collected from a private domestic compost in Montpellier, southern France. Five days after capture, six males and six females were released in an outdoor, shaded plastic cage (11 cages for *D. melanogaster*, 11 cages for *D. suzukii*) (average temperature of 23°C). Each cage contained a live strawberry plant and six organically grown strawberries (Figure S1A). Four fruits were slightly incised to create an artificial wound, and 80 μl of a microbial community isolated from rotten organically grown strawberries were inoculated on the incision for two of these fruits. Two fruits were undamaged. All fruits were surface-sterilized prior to the experiment and had both firm and overripe skin surfaces. After 24 h, eggs were counted on each type of fruit surface (i.e., firm, overripe, wounded, and wounded and inoculated with a microbial community).

##### Assessing male transmission

A gnotobiotic male was released at 8:30 am in a plastic, cylindrical sterile cage (Ø 4 cm, 8 cm high) that contained a blueberry inoculated on the surface with a different bacteria-yeast community (5 000 cells of each microorganism) (Figure 2A). To stimulate male territoriality, and therefore its presence on fruit surface, an axenic female in a small wire mesh box was placed inside the cage. The next day at 8:30 am, the captive axenic female was removed from the cage and a gnotobiotic female associated with a third bacteria-yeast community was released in the cage for 24 h. We recorded whether the male was present or not on the female oviposition site every 1 h 30 min from 9:00 am to 7:30 pm the two days (16 observations in total). The second day, we also recorded mating events every 30 min from 9:00 am to 7:30 pm to determine if the couple mated during the experiment. Fruit manipulation, adult and larval sampling, and experimental conditions were identical to the experiment focused on maternal transmission that is described above. For each *Drosophila* species, we created a cage for every possible female-male-fruit microbial combination among the six microorganisms we studied, i.e., 36 cages. However, some females did not lay eggs. Larvae were therefore collected from 21 cages for *D. melanogaster* and from 27 cages for *D. suzukii*.

Prior to this experiment, two preliminary trials were conducted. First, as both field and laboratory observations suggested that males are largely present on fruits where females can oviposit (Supplemental information), we chose to confirm these observations using small, ecologically realistic microcosms. *D*. *suzukii* adults were collected in a wooded area in Montferrier-sur-Lez, southern France using vinegar baits or were obtained from a laboratory population founded with adults collected in Prades-le-Lez, southern France. *D. melanogaster* adults were all collected from a private domestic compost in Montpellier, southern France. Five days after capture, six males and six females were released in an outdoor, shaded plastic cage (11 cages for *D. melanogaster*, 11 cages for *D. suzukii*) (average temperature of 23°C). Each cage contained a live strawberry plant and six organically grown strawberries of varying condition (see SM1: Figure S1A for an illustration of the experimental system). The following day, we counted the number of females and males on the six fruits (as well as on plant leaves, plant stems, and other parts of the cage) every 30 min from 6:00 am to 10:00 am and from 6:30 pm to 9:30 pm (16 observations) when the flies are most active. Males were frequently and in large numbers observed on fruit. Second, we investigated whether *Drosophila* males deposit extracellular microorganisms on fruit surfaces where females may oviposit. We also wanted to test the existence of a significant link between the time a male spends on such fruit surfaces and the ability of the male to deposit microbial cells. One week-old gnotobiotic *D. suzukii* (population A) or *D. melanogaster* (population C) adults were used. At 8:00 am, two males and one female, each being associated with a different microbial community, were released in a small circular container that contained two blueberries, surface-sterilized and slightly perforated on the peduncle insertion using a sterile pipette tip. A small area around the peduncle insertion was delimited with small spots of orange acrylic paint. One male was gently marked with a small spot of orange acrylic paint on the thorax. We recorded whether each male was present or not on the delimited fruit area every 1 h 30 min from 9:00 am to 7:30 pm (8 observations). At the end of the experiment, we aseptically sampled each fruit area, crushed it with a sterile pestle, and plated (serially diluted) on appropriate solid growth media as described above. For each *Drosophila* species, 27 experimental units were created, i.e., one unit for every possible permutation among the microbial communities associated with the three adults. We measured the male presence on the delimited fruit area for each male on each fruit experimental unit to increase the data resolution (four observations per experimental unit). We found that males can deposit cells of every microbial species studied on the fruit area around the peduncle insertion (Supplemental information). Controls without flies allowed to confirm the absence of cultivable microbial contaminants in our system.

#### Microbial persistence through fly life cycle and over generations

*D. suzukii* (population B) and *D. melanogaster* (population C) fly eggs were collected on grape juice agar plates supplemented with the antifungal cycloheximide (0.1 mL.L^-1^) to help suppressing potential yeasts from the egg surface. Plating egg samples (∼100 eggs per sample, serially diluted in sterile PBS) on solid YPD culture medium, a complete medium for yeast growth, confirmed the absence of cultivable yeasts, and the presence of bacteria. We manually deposited *Drosophila* eggs on halved grape berries, surface-sterilized following the protocol of Behar and colleagues^81^ and placed on sterile vermiculite. Such artificial egg deposition prevented any acquisition of yeasts by the offspring from the faeces of their parents. We then inoculated a suspension of cells of a yeast isolate (20 μL from an overnight culture) on each berry (Figure 3A). Once pupae finished forming, larval fruits were removed. We then aseptically collected five adults of each sex that emerged from pupae on vermiculite within a few hours post emergence. With the remaining adults, we formed heterosexual couples that were placed in new containers containing a halved grape berry previously inoculated with a second yeast isolate (20 μL from an overnight culture). A Petri dish that contained sugar and a piece of sterile hydrated cotton was placed in the cage to ensure fly survival. After five days, the fruit was replaced by another halved grape berry inoculated with a third yeast isolate (20 μL from an overnight culture). After two days, the fruit was replaced by a surface-sterilized grape berry (incised for *D. melanogaster*, intact for *D. suzukii*) for 24 h. We then collected adults individually. Three days later, we collected larvae from the last berry. Adult and larval samples were all individually crushed in sterile PBS for 2 min at 30 Hz with two Ø3 mm sterile glass beads using a Tissue Lyser II (Qiagen). Subsamples (serially diluted) were plated on appropriate solid growth media to detect and discriminate adult- and larvae-associated microorganisms. Plating samples (serially diluted) of surface-sterilized berries not exposed to flies allowed us to confirm the absence of cultivable microorganisms from fruits. The experiment was conducted at 23°C with 65% humidity and a 13.5 h photoperiod.

### Quantification and statistical analysis

All analyses were performed using R 3.6.2.^82^ Larval acquisition of microorganisms, yeast maintenance throughout metamorphosis, and yeast maintenance and acquisition in mature adults were all analysed using generalized linear mixed models with binomial distribution and logit function (package *lme4* v1.1-27.1^83^: function *glmer*). Each of these variables was measured as the proportion of successful detection of a microorganism of interest (i.e., inoculated at the beginning of the experiment) among insect samples (see Supplemental information for a summary of the corresponding variables measured as the number of cells of a microorganism of interest among insect samples). ‘*Experimental unit*’ information (i.e., cage identity) was included as a random factor in each model. Backward stepwise selection allowed us to remove non-significant variables from initial full models (α = 0.05). Multiple contrasts were used to detect significant differences between factor levels when appropriate (package *emmeans* v1.6.2-1^84^: function *emmeans* with Tukey adjustment).

In the first experiment, focusing on maternal transmission, larval acquisition of microorganisms was analysed with ‘*Microbial origin*’, ‘*Microbial species’*, ‘*Drosophila species*’, their three 2-way interactions, ‘*Number of larvae collected*’, and ‘*Concentration of fruit microorganisms*’ modelled as fixed factors (Table S2).

In the second experiment, which included male transmission, larval acquisition of microorganisms was analysed with ‘*Microbial origin*’, ‘*Microbial species*, ‘*Drosophila species*’, their three 2-way interactions, and ‘*Number of larvae collected*’ modelled as fixed factors (Table S5). To investigate the mechanisms behind male transmission, transmission rates from males were analysed with the fixed factors ‘*Microbial species*, ‘*Drosophila species*’, their 2-way interaction, ‘*Number of larvae collected*’, ‘*Male presence on oviposition site’*, and ‘*Mating status*’ (Table S5).

To test the repeatability of strain transmission potential among experiments, rates of larval acquisition of microorganisms from females and fruit (not from males) were analysed with ‘*Microbial origin*’, ‘*Microbial species*, ‘*Drosophila species*’, ‘*Experiment identity*’, the three 2-way interactions between ‘*Experiment identity*’ and the aforementioned variables, and ‘*Number of larvae collected*’ modelled as fixed factors (Table S6).

For the two experiments presented above, the interactions between microbiota members were not taken into account in our models. While adults and fruits were always co-inoculated with yeasts and bacteria, our experimental design was not designed to unravel any synergistic or antagonistic microbial interactions and their effects on microbial transmission or maintenance. This would have necessitated numerous replicates of each combination. Our goal rather was to get closer to symbiotic conditions in the wild, and therefore to unrealistic mono-associations. Note, however, that interactions between microbiota members are common and can impact microbial nutrition,^85^ multiplication and transmission,^52^^,^^78^ as well as host nutrition.^86^

In the third experiment, focusing on yeast persistence through fly life cycle and over generations, yeast maintenance through fly metamorphosis was analysed with ‘*Yeast species*’, ‘*Drosophila species*’, and ‘*Drosophila sex*’ modelled as fixed factors. Presence of larvae-associated yeasts, first environmental yeasts, and second environmental yeasts in mature adults were analysed separately using the same three fixed factors as above (Table S7). Presence of first and second environmental yeasts in mature adults were compared using ‘*Order of the environmental yeast*’ (i.e., first or second) as a fixed factor in addition to ‘*Yeast species*’, ‘*Drosophila species*’, and ‘*Drosophila sex*’.

## Data Availability

• Datasets are available in the open data repository Zenodo (https://doi.org/10.5281/zenodo.6481191). • This paper does not report original code. • Any additional information required to reanalyze the data reported in this paper is available from the lead contact upon request.
