# Supplementary material for: Microbiota acquisition and transmission in Drosophila flies
Source: iScience. 2023 Aug 17;26(9):107656. doi: 10.1016/j.isci.2023.107656 (PMC10475513; doi:10.1016/j.isci.2023.107656)
Supplement: Document S1. Figures S1–S6 and Tables S2 and S5–S9 [file mmc1.pdf]

iScience, Volume *26*

Supplemental information

Microbiota acquisition and transmission in *Drosophila* flies

Robin Guilhot, Anne Xuéreb, Auxane Lagmairi, Laure Olazcuaga, and Simon Fellous

SM 1. *D. melanogaster* and *D. suzukii* females preferentially oviposit on different fruit substrates.

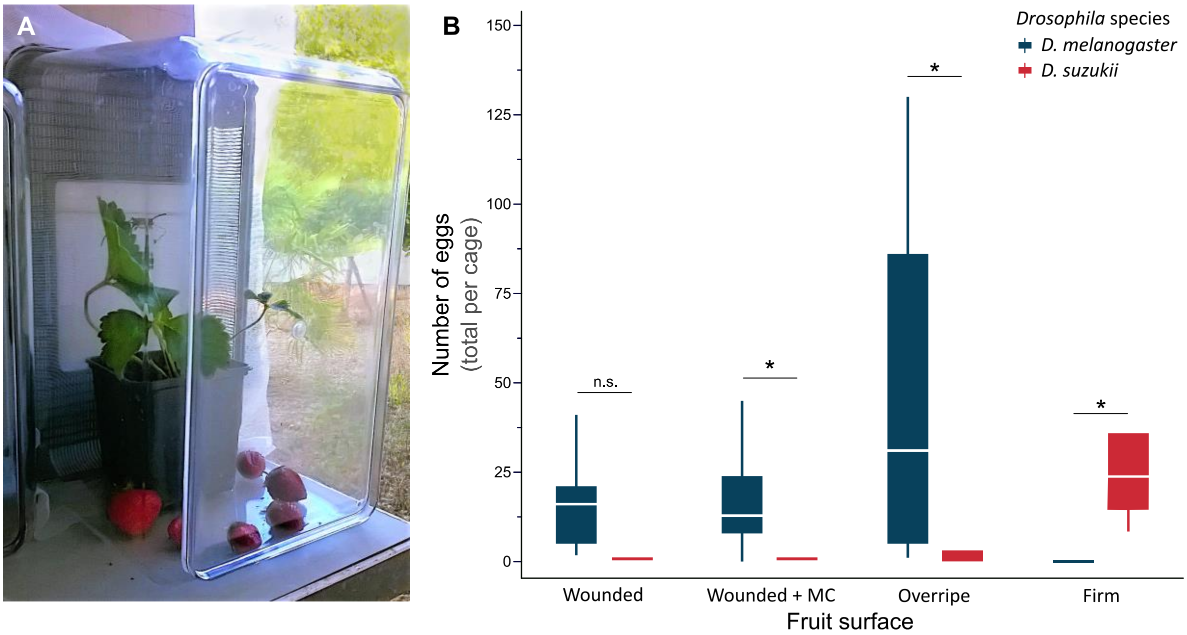

Figure S1. *D. melanogaster* and *D. suzukii* females preferentially oviposit on different fruit substrates. Related to Figure 1. (A) Illustration of the experimental system (picture credit: R. Guilhot). (B) Boxplot of the number of eggs laid on each fruit surface. MC means Microbial Community. The asterisk '*' indicates a significant difference ($\alpha = 0.05$). *D. melanogaster* females laid more eggs, than *D. suzukii* females, on wounded and inoculated with a microbial community fruit surfaces ($\chi^2 = 8.45$, df = 1, p = 0.0036) as well as on overripe fruit surfaces ($\chi^2 = 30.15$, df = 1, p < 0.0001). On the contrary, *D. suzukii* females laid more eggs on firm fruit surfaces than *D. melanogaster* females ($\chi^2 = 26.38$, df = 1, p < 0.0001). Overall, the number of eggs deposited on wounded fruit surfaces did not significantly differ between the two fly species ($\chi^2 = 3.16$, df = 1, p = 0.0754) (GLMM with Poisson distribution and Log link function, fixed effects: '*Drosophila species*', '*Type of fruit surface*', their interaction and '*Experimental block*'). Wild-caught and laboratory *D. suzukii* females (five and six cages respectively) did not differ in the number of eggs they laid on the different fruit substrates ($\chi^2 = 4.76$, df = 3, p = 0.1902) (GLMM with Poisson distribution, fixed effects: '*Drosophila population*', '*Type of fruit surface*', their interaction and '*Experimental block*').

SM 2. Analysis of maternal transmission and acquisition of fruit microorganisms by larvae.

Table S2. Response of larval acquisition of microorganisms to different factors. Related to Figure 1. Generalized linear mixed models (GLMM) with binomial distribution and logit link function ($\alpha = 0.05$). P-values are coded such as: $p < 0.05$*, $p < 0.01$**, $p < 0.001$***. Variables followed by a single letter were removed from the initial full model (backward stepwise selection), in an order indicated by the alphabetic order of the letter (i.e. variable (a) was the first removed from the initial full model).

**Using Microbial species as a fixed factor**	
Microbial origin	$\chi^2 = 6.02$, df = 1, p = 0.0142*
Microbial species	$\chi^2 = 43.90$, df = 5, p < 0.0001***
Drosophila species	$\chi^2 = 0.21$, df = 1, p = 0.6438
Microbial origin × Microbial species (b)	$\chi^2 = 1.37$, df = 5, p = 0.9276
Microbial origin × *Drosophila* species (d)	$\chi^2 = 3.26$, df = 1, p = 0.0709
Microbial species × *Drosophila* species (c)	$\chi^2 = 7.71$, df = 5, p = 0.1732
Concentration of fruit microorganisms (a)	$\chi^2 = 0.18$, df = 1, p = 0.6734
Number of larvae collected	$\chi^2 = 8.30$, df = 1, p = 0.004**

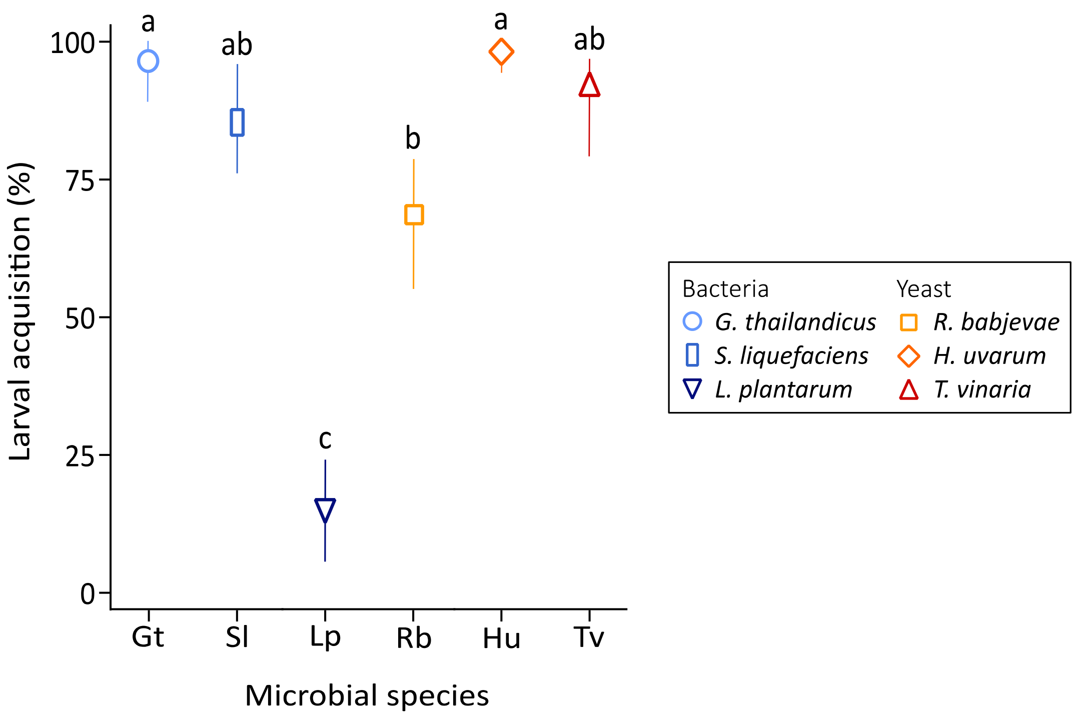

Figure S2. Proportion of larval acquisition of the six microorganisms, independent of their origin or the *Drosophila* species studied (% larval samples). Related to Figure 1. Symbols and related bars indicate mean and 95% CI (calculated using normal approximation method) for each microorganism. Different letters indicate statistically significant differences (α = 0.05).

SM 3. Prerequisites to male transmission of extracellular microorganisms to larvae: *Drosophila* males are present on fruits where females are susceptible to lay eggs.

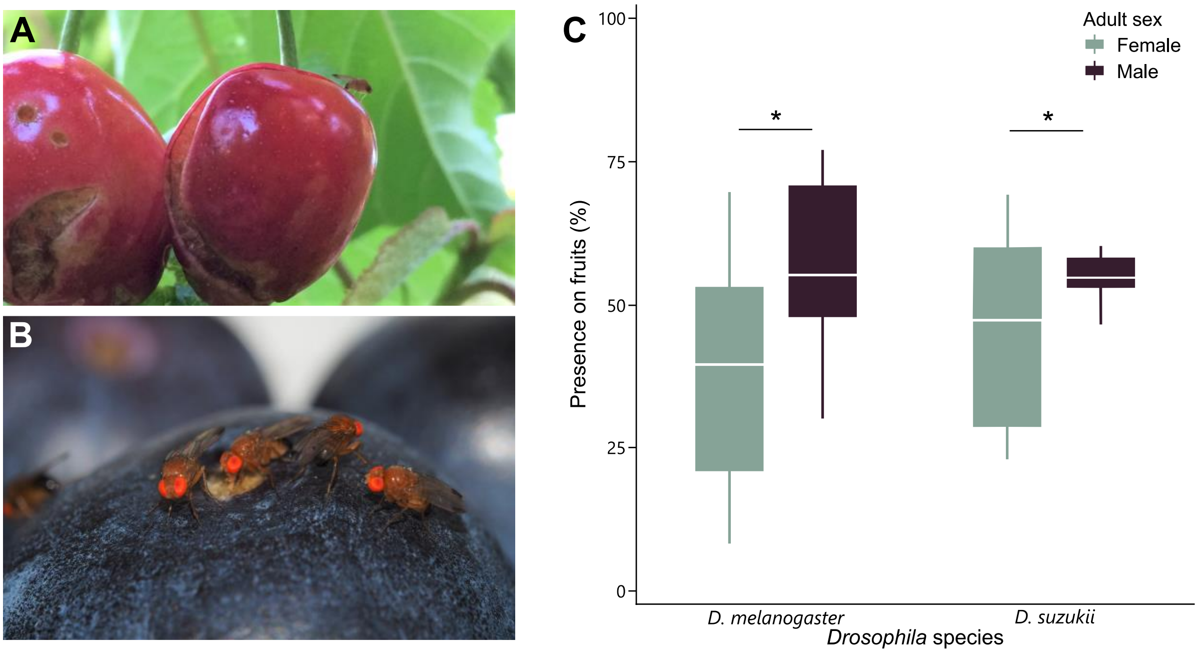

Figure S3. *Drosophila* males are present on fruits where females are susceptible to lay eggs. Related to Figure 2. (A) A wild *D. suzukii* male on a ripe cherry (picture credit: S. Fellous). (B) *D. suzukii* male aggregation on a blueberry in the laboratory (picture credit: R. Guilhot). (C) Boxplot of the total percentage of *Drosophila* males and females present in the cage that were observed on fruit. The asterisk '*' indicates a significant difference ($\alpha = 0.05$). Males were frequently and in large numbers observed on fruit. *D. melanogaster* males were more present than females on fruits ($\chi^2 = 8.11$, df = 1, p = 0.0044), as well as *D. suzukii* males ($\chi^2 = 4.21$, df = 1, p = 0.0402) (GLMM for each fly species with Poisson distribution and Log link function, fixed factors: '*Drosophila sex*' and '*Experimental block*').

SM 4. Prerequisites to male transmission of extracellular microorganisms to larvae: *Drosophila* males deposit extracellular microorganisms on fruit sites where females are likely to lay eggs.

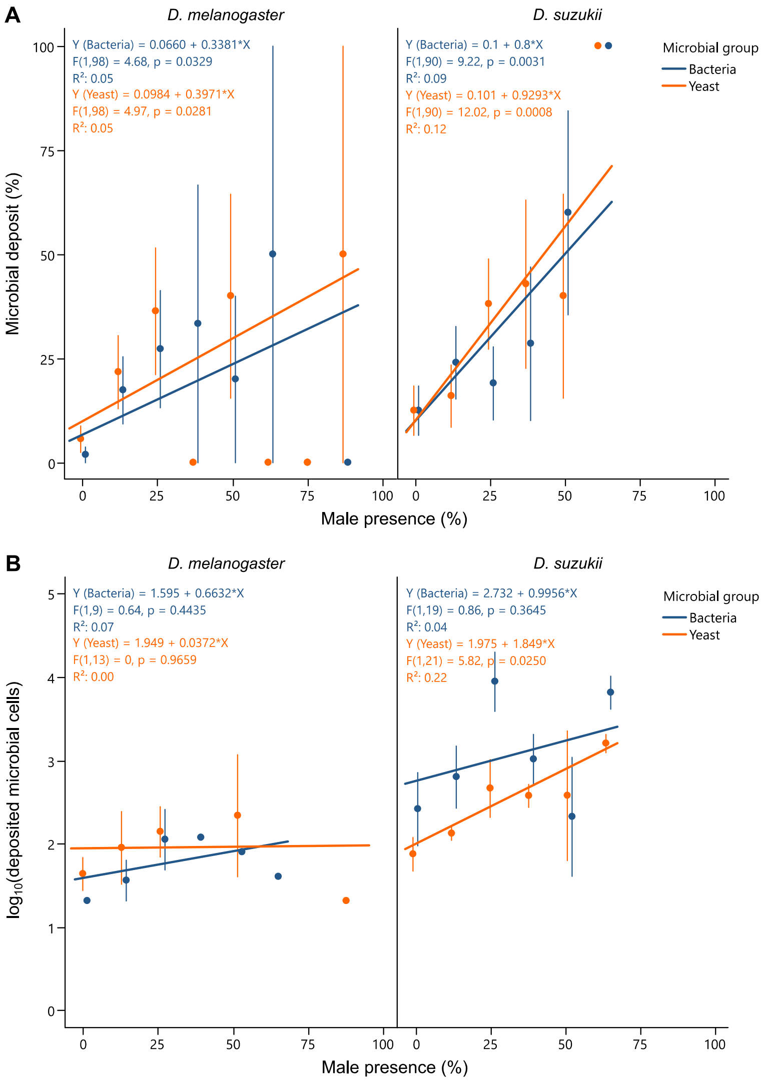

Figure S4. *Drosophila* males deposit extracellular microorganisms on fruit sites where females are likely to lay eggs. Related to Figure 2. (A) Linear regression of deposition of microbial cells on percentage of male presence on the delimited fruit area. A positive relationship between the time spent by a male on an observed fruit area and the likelihood of retrieving the microorganisms it carried is suggested by our results. (B) Linear regression of quantity of microbial cells counted from fruit sample on percentage of male presence on the delimited fruit area. The quantity of microbial cells on fruit surface did not correlate, or only weakly, with the time spent by males in the corresponding area. Multiplication of microorganisms on the fruit flesh after the first male's deposit, independent of the next male's deposit(s), could explain this lack of relationship. Data points indicate mean ± SEM.

SM 5. Analysis of male transmission, maternal transmission, and acquisition of fruit microorganisms by larvae.

Table S5. Response of larval microbial acquisition to different factors. Related to Figure 2. Generalized linear mixed models (GLMM) with binomial distribution and logit link function. P-values are coded such as: $p < 0.05$*, $p < 0.01$**, $p < 0.001$***. Variables followed by a single letter were removed from the initial full model (backward stepwise selection), in an order indicated by the alphabetic order of the letter (i.e. variable (a) was the first removed from the initial full model).

**(A) Response of larval microbial acquisition to different factors**	
Microbial origin	$\chi^2 = 10.07$, df = 2, p = 0.0065**
Microbial species	$\chi^2 = 68.45$, df = 8, p < 0.0001***
Drosophila species	$\chi^2 = 13.06$, df = 3, p = 0.0045**
Microbial origin × Microbial species (b)	$\chi^2 = 8.59$, df = 10, p = 0.5715
Microbial origin × *Drosophila* species (a)	$\chi^2 = 0.13$, df = 2, p = 0.938
Microbial species × *Drosophila* species	$\chi^2 = 12.05$, df = 5, p = 0.0342*
Number of larvae collected	$\chi^2 = 0.01$, df = 1, p = 0.9101
**(B) Response of larval acquisition of male microorganisms to different factors**	
Microbial species (e)	$\chi^2 = 4.75$, df = 5, p = 0.4477
Drosophila species	$\chi^2 = 7.24$, df = 1, p = 0.0071**
Microbial species × *Drosophila* species (a)	$\chi^2 = 0.57$, df = 5, p = 0.9894
Male presence on oviposition site (% observations) (c)	$\chi^2 \approx 0$, df = 1, p = 0.9887
Mating status (not mated/mated during the experiment) (d)	$\chi^2 = 0.03$, df = 1, p = 0.8564
Number of larvae collected (b)	$\chi^2 \approx 0$, df = 1, p = 0.9878

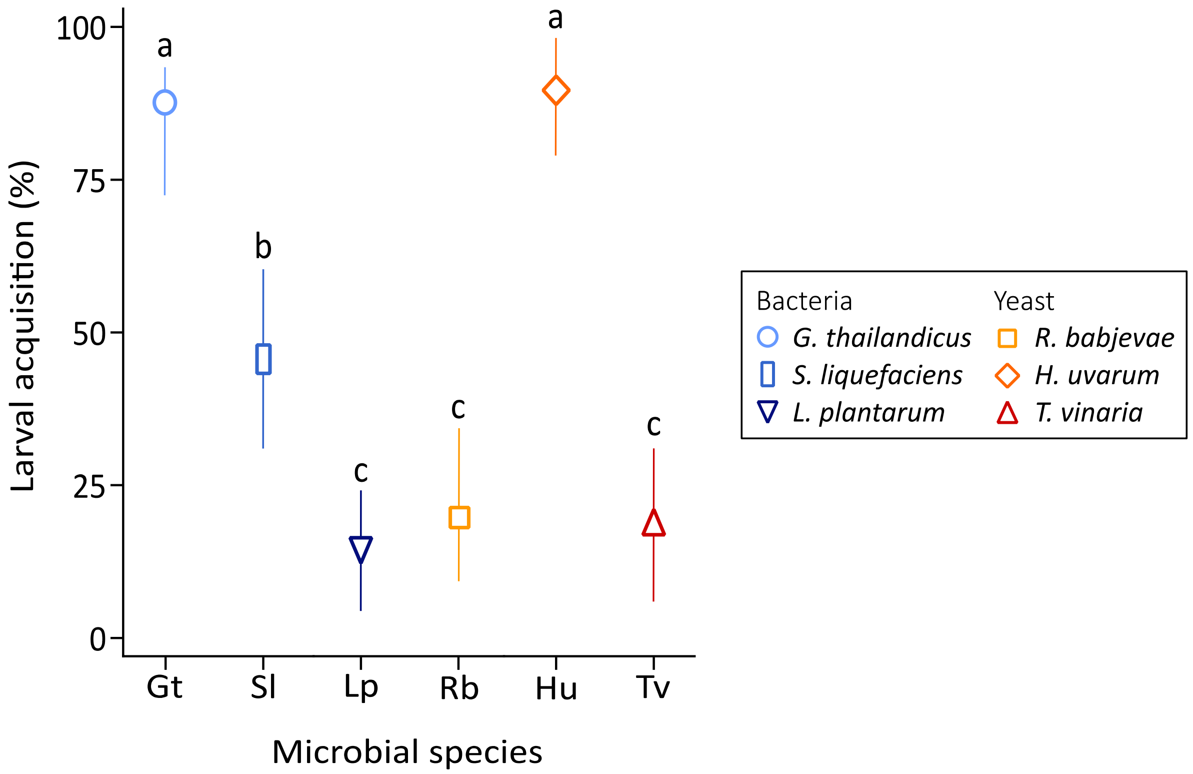

Figure S5. Proportion of larval acquisition of the six microorganisms, independent of their origin or the *Drosophila* species studied (% larval samples). Related to Figure 2. Symbols and related bars indicate mean and 95% CI (calculated using normal approximation method) for each microorganism. Different letters indicate statistically significant differences (α = 0.05).

SM 6. Investigating influence of the experimental context on larval acquisition of maternal and fruit microorganisms.

Table S6. Response of larval microbial acquisition to different factors, including the experiment identity. Related to Figures 1 and 2. Generalized linear mixed models (GLMM) with binomial distribution and logit link function. P-values are coded such as: $p < 0.05$*, $p < 0.01$**, $p < 0.001$***. Variables followed by a single letter were removed from the initial full model (backward stepwise selection), in an order indicated by the alphabetic order of the letter (i.e. variable (a) was the first removed from the initial full model).

**Using Microbial species as a fixed factor**	
Microbial origin	$\chi^2 = 6.28$, df = 1, $p = 0.0122$*
Microbial species	$\chi^2 = 64.03$, df = 5, $p < 0.0001$***
Drosophila species	$\chi^2 = 2.15$, df = 1, $p = 0.1427$
Experiment	$\chi^2 = 25.01$, df = 1, $p < 0.0001$***
Experiment × Microbial origin (b)	$\chi^2 = 0.96$, df = 1, $p = 0.3272$
Experiment × Microbial species	$\chi^2 = 16.38$, df = 5, $p = 0.0058$**
Experiment × *Drosophila* species (a)	$\chi^2 = 0.33$, df = 1, $p = 0.5676$
Number of larvae collected	$\chi^2 = 5.80$, df = 1, $p = 0.0161$*

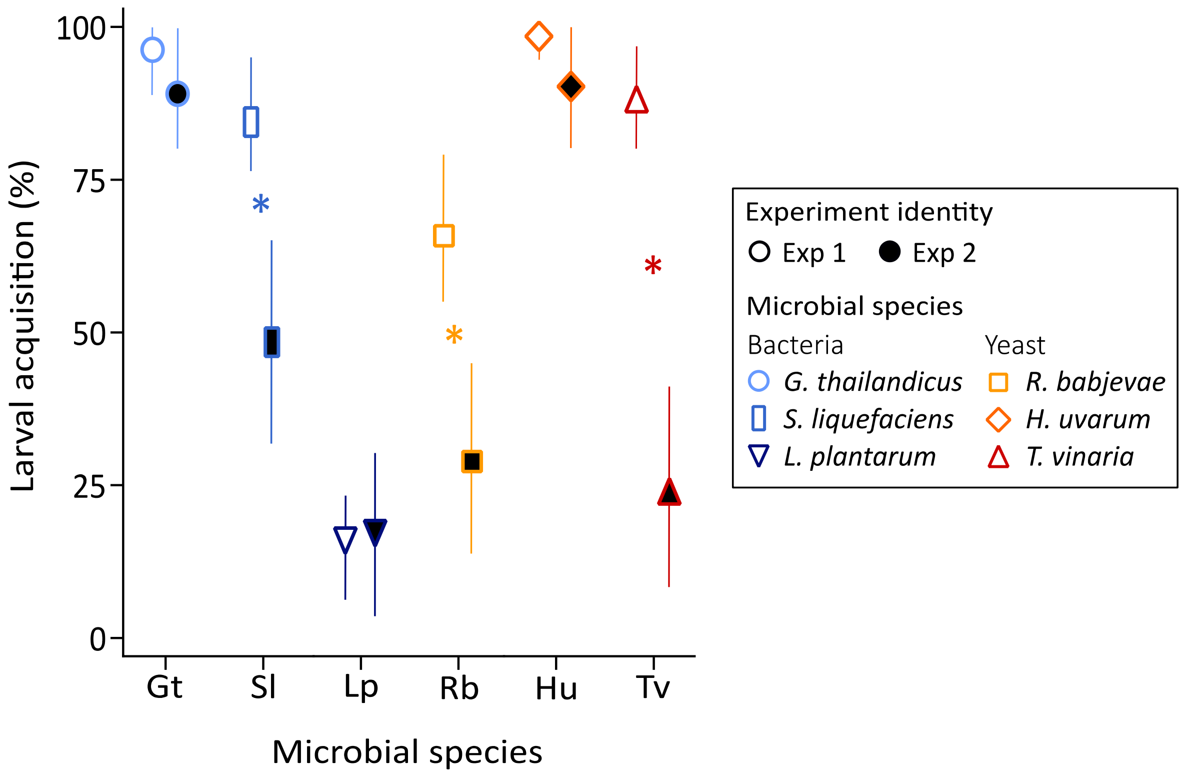

Figure S6. Proportion of larval acquisition of the six microorganisms in the two experiments focusing on the origin of larval associated microorganisms, independent of the microbial origin or the *Drosophila* species studied (% larval samples). Related to Figures 1 and 2. Symbols and related bars indicate mean and 95% CI (calculated using normal approximation method) for each microorganism. Asterisks '*' indicate statistically significant differences (α = 0.05).

SM 7. Analysis of the presence of yeasts of different origins in mature *Drosophila* adults.

Table S7. Response of the presence of yeasts of different origins in mature *Drosophila* adults to different factors. Related to Figure 3. Generalized linear mixed models (GLMM) with binomial distribution and logit link function. P-values are coded such as: $p < 0.05$*, $p < 0.01$**, $p < 0.001$***. Variables followed by a single letter were removed from the initial full model (backward stepwise selection), in an order indicated by the alphabetic order of the letter (i.e. variable (a) was the first removed from the initial full model).

**(A) Presence of larvae-associated yeast**	
Yeast species	$\chi^2 = 3.01$, df = 2, p = 0.2224
Drosophila species (a)	$\chi^2 = 0.05$, df = 1, p = 0.8316
Drosophila sex	$\chi^2 = 0.76$, df = 1, p = 0.3845
**(B) Presence of first environmental yeast**	
Yeast species	$\chi^2 = 5.96$, df = 2, p = 0.0509
Drosophila species (a)	$\chi^2 \approx 0$, df = 1, p ≈ 1
Drosophila sex	$\chi^2 = 8.90$, df = 1, p = 0.0028**
**(C) Presence of second environmental yeast**	
Yeast species	$\chi^2 = 7.98$, df = 2, p = 0.0185*
Drosophila species	$\chi^2 = 0.62$, df = 1, p = 0.431
Drosophila sex (a)	$\chi^2 = 0.27$, df = 1, p = 0.604

SM 8. Microbial isolates used in the present study: identity, source, growth conditions, and mention in relevant literature.

Table S8. Microbial isolates used in the present study: identity, source, growth conditions, and mention in relevant literature. Related to STAR Methods.

Microorganism	Source	Growth medium	Mention in relevant literature (species or genus level)
**BACTERIA**			
Gluconobacter thailandicus	[1] Isolated from a fly-infested grape berry	Mannitol (MAN) - 24°C	[2,3,4,5,6,7,8,9]
Serratia liquefaciens	[1] Isolated from *D. suzukii* ovaries	MAN - 24°C	[2,4,6,10]
Lactobacillus plantarum	[11] (GenBank #EU096230) Isolated from *D. melanogaster*	De Man, Rogosa and Sharpe (MRS) - 30°C	[2,3,4,6,7,10,11,12,13,14,15]
**YEASTS**			
Rhodotorula babjevae	[16] (GenBank #MN684819) Isolated from a fly-infested grape berry	Yeast Extract-Peptone-Dextrose (YPD) - 24°C	[17,18,19,20]
Hanseniaspora uvarum	[16] (GenBank #MN684824) Isolated from *D. melanogaster* feces	YPD - 24°C	[8,17,19,20,21,22,23,24,25,26]
Trigonopsis vinaria	[16] (GenBank #MN684816) Isolated from *D. suzukii* ovaries	YPD - 24°C	[17,27] Initially described as *Candida vinaria* from grape must (Japan), rarely isolated

SM 9. Mean number of symbionts per fly sample.

Table S9. Log10(Number of symbiont cells+1) per sample. Related to Figures 1C, 2C, 3 and STAR Methods. Mean ± standard error around the mean.

(A) Log10(Number of symbiont cells+1) per larval sample (see Figure 1C)

	Maternal transmission		Acquisition of fruit microorganisms	
	D. melanogaster	*D. suzukii*	*D. melanogaster*	*D. suzukii*
G. thailandicus	4.26 ± 0.18 (n=13)	4.57 ± 0.23 (n=11)	4.59 ± 0.23 (n=16)	3.71 ± 0.48 (n=15)
S. liquefaciens	3.22 ± 0.40 (n=12)	2.83 ± 0.45 (n=16)	3.54 ± 0.20 (n=11)	2.45 ± 0.40 (n=12)
L. plantarum	0.15 ± 0.15 (n=15)	0.40 ± 0.24 (n=15)	0.64 ± 0.34 (n=13)	0.21 ± 0.14 (n=15)
R. babjevae	0.67 ± 0.24 (n=16)	1.75 ± 0.39 (n=17)	2.62 ± 0.22 (n=12)	2.10 ± 0.43 (n=14)
H. uvarum	3.83 ± 0.29 (n=10)	4.49 ± 0.24 (n=12)	4.47 ± 0.17 (n=15)	4.15 ± 0.32 (n=17)
T. vinaria	3.24 ± 0.27 (n=14)	1.83 ± 0.45 (n=13)	3.54 ± 0.38 (n=13)	3.10 ± 0.35 (n=11)
Overall mean	2.37 ± 0.21 (n=80)	2.49 ± 0.22 (n=84)	3.31 ± 0.19 (n=80)	2.64 ± 0.21 (n=84)

(B) Log10(Number of symbiont cells+1) per larval sample (see Figure 2C)

	Male transmission		Maternal transmission		Acquisition of fruit microorganisms	
	D. melanogaster	*D. suzukii*	*D. melanogaster*	*D. suzukii*	*D. melanogaster*	*D. suzukii*
G. thailandicus	5.41 ± 0.09 (n=9)	1.44 ± 0.61 (n=9)	4.29 ± 0.58 (n=4)	4.71 ± 0.68 (n=8)	4.83 ± 0.32 (n=8)	3.86 ± 0.73 (n=10)
S. liquefaciens	3.12 ± 0.85 (n=5)	0.29 ± 0.29 (n=8)	3.57 ± 0.68 (n=9)	1.34 ± 0.61 (n=11)	1.93 ± 0.76 (n=7)	0.78 ± 0.61 (n=8)
L. plantarum	0 (n=7)	0.41 ± 0.28 (n=10)	0 (n=8)	0.17 ± 0.17 (n=8)	1.20 ± 0.54 (n=6)	0.44 ± 0.44 (n=9)
R. babjevae	0 (n=4)	0 (n=9)	0.13 ± 0.13 (n=10)	0.71 ± 0.47 (n=9)	0.73 ± 0.47 (n=7)	1.49 ± 0.48 (n=9)
H. uvarum	3.85 ± 0.18 (n=8)	3.26 ± 0.78 (n=8)	4.13 ± 0.49 (n=7)	3.19 ± 0.64 (n=11)	4.34 ± 0.45 (n=6)	3.81 ± 0.68 (n=8)
T. vinaria	0.82 ± 0.54 (n=9)	0 (n=10)	2.15 ± 1.24 (n=4)	0.51 ± 0.51 (n=7)	1.01 ± 0.66 (n=8)	0.59 ± 0.46 (n=10)
Overall mean	2.44 ± 0.36 (n=42)	0.84 ± 0.22 (n=54)	2.10 ± 0.35 (n=42)	1.83 ± 0.31 (n=54)	2.35 ± 0.34 (n=42)	1.83 ± 0.30 (n=54)

(C) Log10(Number of yeast cells+1) per young adult (see Figure 3B)

	Maintenance through metamorphosis	
	D. melanogaster	*D. suzukii*
R. babjevae	0.86 ± 0.45 (n=10)	0.81 ± 0.45 (n=10)
H. uvarum	0.56 ± 0.38 (n=9)	0 (n=10)
T. vinaria	2.03 ± 0.47 (n=10)	1.12 ± 0.62 (n=10)
Overall mean	1.17 ± 0.27 (n=29)	0.65 ± 0.26 (n=30)

(D) Log10(Number of yeast cells+1) per mature adult (see Figure 3C)

	Presence of larvae-associated yeast		Acquisition of 1st environmental yeast		Acquisition of 2nd environmental yeast	
	D. melanogaster	*D. suzukii*	*D. melanogaster*	*D. suzukii*	*D. melanogaster*	*D. suzukii*
R. babjevae	1.20 ± 0.33 (n=14)	1.38 ± 0.74 (n=6)	1.89 ± 0.35 (n=12)	2.82 ± 0.25 (n=4)	0 (n=14)	0 (n=6)
H. uvarum	3.04 ± 0.39 (n=18)	1.43 ± 0.83 (n=4)	3.77 ± 0.31 (n=10)	3.21 ± 0.15 (n=6)	3.15 ± 0.30 (n=12)	1.43 ± 0.84 (n=6)
T. vinaria	0 (n=8)	1.13 ± 0.65 (n=6)	1.70 ± 0.25 (n=18)	1.47 ± 0.50 (n=6)	0.20 ± 0.13 (n=14)	1.00 ± 1.00 (n=4)
Overall mean	1.90 ± 0.30 (n=40)	1.30 ± 0.39 (n=16)	2.30 ± 0.22 (n=40)	2.41 ± 0.29 (n=16)	0.93 ± 0.27 (n=40)	0.79 ± 0.41 (n=16)

(E) Log10(Number of yeast cells+1) per larval sample (see Figure 3D)

	Transmission of larvae-	Transmission of 1st	Transmission of 2nd

	associated yeast	environmental yeast	environmental yeast
	D. melanogaster	*D. melanogaster*	*D. melanogaster*
R. babjevae	0.61 ± 0.43 (n=7)	0.80 ± 0.62 (n=6)	0 (n=7)
H. uvarum	5.67 ± 0.17 (n=9)	5.41 ± 0.17 (n=5)	2.91 ± 1.20 (n=6)
T. vinaria	4.13 ± 2.12 (n=4)	3.09 ± 0.75 (n=9)	0 (n=7)
Overall mean	3.49 ± 0.70 (n=20)	2.81 ± 0.58 (n=20)	0.97 ± 0.52 (n=20)

REFERENCES

1. Guilhot, R., Fellous, S., & Cohen, J. E. (2020). Yeast facilitates the multiplication of Drosophila bacterial symbionts but has no effect on the form or parameters of Taylor's law. *PloS one, 15(11),* e0242692. https://doi.org/10.1371/journal.pone.0242692
2. Corby-Harris, V., Pontaroli, A. C., Shimkets, L. J., Bennetzen, J. L., Habel, K. E., & Promislow, D. E. (2007). Geographical distribution and diversity of bacteria associated with natural populations of Drosophila melanogaster. *Applied and environmental microbiology, 73(11),* 3470-3479. https://doi.org/10.1128/AEM.02120-06
3. Fink, C., Staubach, F., Kuenzel, S., Baines, J. F., & Roeder, T. (2013). Noninvasive analysis of microbiome dynamics in the fruit fly Drosophila melanogaster. *Applied and environmental microbiology, 79(22),* 6984-6988. https://doi.org/10.1128/AEM.01903-13
4. Staubach, F., Baines, J. F., Künzel, S., Bik, E. M., & Petrov, D. A. (2013). Host species and environmental effects on bacterial communities associated with Drosophila in the laboratory and in the natural environment. *PloS one, 8(8),* e70749. https://doi.org/10.1371/journal.pone.0070749
5. Rombaut, A., Guilhot, R., Xuéreb, A., Benoit, L., Chapuis, M. P., Gibert, P., & Fellous, S. (2017). Invasive Drosophila suzukii facilitates Drosophila melanogaster infestation and sour rot outbreaks in the vineyards. *Royal Society open science, 4(3),* 170117. https://doi.org/10.1098/rsos.170117
6. Vacchini, V., Gonella, E., Crotti, E., Prosdocimi, E. M., Mazzetto, F., Chouaia, B., Callegari, M., Mapelli, F., Mandrioli, M., Alma, A., & Daffonchio, D. (2017). Bacterial diversity shift determined by different diets in the gut of the spotted wing fly Drosophila suzukii is primarily reflected on acetic acid bacteria. *Environmental microbiology reports, 9(2),* 91-103. https://doi.org/10.1111/1758-2229.12505
7. Pais, I. S., Valente, R. S., Sporniak, M., & Teixeira, L. (2018). Drosophila melanogaster establishes a species-specific mutualistic interaction with stable gut-colonizing bacteria. *PloS biology, 16(7)*. https://doi.org/10.1371/journal.pbio.2005710
8. Solomon, G. M., Dodangoda, H., McCarthy-Walker, T., Ntim-Gyakari, R., & Newell, P. D. (2019). The microbiota of Drosophila suzukii influences the larval development of Drosophila melanogaster. *PeerJ, 7,* e8097. https://doi.org/10.7717/peerj.8097
9. Wang, Y., Kapun, M., Waidele, L., Kuenzel, S., Bergland, A. O., & Staubach, F. (2020). Common structuring principles of the Drosophila melanogaster microbiome on a continental scale and between host and substrate. *Environmental microbiology reports, 12(2),* 220-228. https://doi.org/10.1111/1758-2229.12826

10. Chandler, J. A., Lang, J. M., Bhatnagar, S., Eisen, J. A., & Kopp, A. (2011). Bacterial communities of diverse Drosophila species: ecological context of a host–microbe model system. *PloS genet, 7(9),* e1002272. https://doi.org/10.1371/journal.pgen.1002272
11. Ryu, J. H., Kim, S. H., Lee, H. Y., Bai, J. Y., Nam, Y. D., Bae, J. W., Lee, D. G., Shin, S. C., Ha, E. M., & Lee, W. J. (2008). Innate immune homeostasis by the homeobox gene caudal and commensal-gut mutualism in Drosophila. *Science, 319(5864),* 777-782. https://doi.org/10.1126/science.1149357
12. Ren, C., Webster, P., Finkel, S. E., & Tower, J. (2007). Increased internal and external bacterial load during Drosophila aging without life-span trade-off. *Cell metabolism, 6(2),* 144-152. https://doi.org/10.1016/j.cmet.2007.06.006
13. Wong, A. C. N., Ng, P., & Douglas, A. E. (2011). Low-diversity bacterial community in the gut of the fruitfly Drosophila melanogaster. *Environmental microbiology, 13(7),* 1889-1900. https://doi.org/10.1111/j.1462-2920.2011.02511.x
14. Obadia, B., Güvener, Z. T., Zhang, V., Ceja-Navarro, J. A., Brodie, E. L., Ja, W. W., & Ludington, W. B. (2017). Probabilistic Invasion Underlies Natural Gut Microbiome Stability. *Current Biology, 27(13),* 1999-2006. https://doi.org/10.1016/j.cub.2017.05.034
15. Erkosar, B., Yashiro, E., Zajitschek, F., Friberg, U., Maklakov, A. A., van der Meer, J. R., & Kawecki, T. J. (2018). Host diet mediates a negative relationship between abundance and diversity of Drosophila gut microbiota. *Ecology and evolution, 8(18),* 9491-9502. https://doi.org/10.1002/ece3.4444
16. Guilhot, R., Xuéreb, A., & Fellous, S. (2020) The partitioning of symbionts effects on host resource acquisition and developmental plasticity. *bioRxiv.* https://doi.org/10.1101/2020.04.27.064667
17. Lachance, M. A., Gilbert, D. G., & Starmer, W. T. (1995). Yeast communities associated with Drosophila species and related flies in an eastern oak-pine forest: a comparison with western communities. *Journal of industrial microbiology and biotechnology, 14(6),* 484-494. https://doi.org/10.1007/BF01573963
18. Renouf, V., Claisse, O., & Lonvaud-Funel, A. (2005). Understanding the microbial ecosystem on the grape berry surface through numeration and identification of yeast and bacteria. *Australian Journal of Grape and Wine Research, 11(3),* 316-327. https://doi.org/10.1111/j.1755-0238.2005.tb00031.x
19. Barata, A., Santos, S. C., Malfeito-Ferreira, M., & Loureiro, V. (2012). New insights into the ecological interaction between grape berry microorganisms and Drosophila flies during the development of sour rot. *Microbial ecology, 64(2),* 416-430. https://doi.org/10.1007/s00248-012-0041-y
20. Bellutti, N., Gallmetzer, A., Innerebner, G., Schmidt, S., Zelger, R., & Koschier, E. H. (2018). Dietary yeast affects preference and performance in Drosophila suzukii. *Journal of pest science, 91(2),* 651-660. ttps://doi.org/10.1007/s10340-017-0932-2

21. Chandler, J. A., Eisen, J. A., & Kopp, A. (2012). Yeast communities of diverse Drosophila species: comparison of two symbiont groups in the same hosts. *Applied and environmental microbiology, 78(20),* 7327-7336. https://doi.org/10.1128/AEM.01741-12

22. Hamby, K.A., Hernández, A., Boundy-Mills, K., & Zalom, F. G. (2012). Associations of yeasts with spotted-wing Drosophila (Drosophila suzukii; Diptera: Drosophilidae) in cherries and raspberries. *Applied and environmental microbiology, 78(14),* 4869-4873. https://doi.org/10.1128/AEM.00841-12

23. Stamps, J. A., Yang, L. H., Morales, V. M., & Boundy-Mills, K. L. (2012). Drosophila regulate yeast density and increase yeast community similarity in a natural substrate. *PloS one, 7(7),* e42238. https://doi.org/10.1371/journal.pone.0042238

24. Hoang, D., Kopp, A., & Chandler, J. A. (2015). Interactions between Drosophila and its natural yeast symbionts—Is Saccharomyces cerevisiae a good model for studying the fly-yeast relationship?. *PeerJ, 3,* e1116. https://doi.org/10.7717/peerj.1116

25. Lam, S. S., & Howell, K. S. (2015). Drosophila-associated yeast species in vineyard ecosystems. *FEMS microbiology letters, 362(20),* fnv170. https://doi.org/10.1093/femsle/fnv170

26. Quan, A. S., & Eisen, M. B. (2018). The ecology of the Drosophila-yeast mutualism in wineries. *PloS one, 13(5),* e0196440. https://doi.org/10.1371/journal.pone.0196440

27. Quirós, M., Rojas, V., Gonzalez, R., & Morales, P. (2014). Selection of non-Saccharomyces yeast strains for reducing alcohol levels in wine by sugar respiration. *International Journal of Food Microbiology, 181,* 85-91. https://doi.org/10.1016/j.ijfoodmicro.2014.04.024
